# ﻿Three new species of *Elatostema* (Urticaceae) from Thailand

**DOI:** 10.3897/phytokeys.215.94591

**Published:** 2022-12-13

**Authors:** Natthawut Triyutthachai, Long-Fei Fu, Pramote Triboun, Yi-Gang Wei, Pimwadee Pornpongrungrueng

**Affiliations:** 1 Applied Taxonomic Research Center, Department of Biology, Faculty of Science, Khon Kaen University, Khon Kaen 40002, Thailand Khon Kaen University Khon Kaen Thailand; 2 Guangxi Key Laboratory of Plant Conservation and Restoration Ecology in Karst Terrain, Guangxi Institute of Botany, Guangxi Zhuang Autonomous Region and Chinese Academy of Sciences, Guilin, 541006, China Guangxi Institute of Botany, Guangxi Zhuang Autonomous Region and Chinese Academy of Sciences Guilin China; 3 National Biobank of Thailand, National Science and Technology Development Agency, Thailand Science Park, Khlong Nueng, Khlong Luang, Pathum Thani 12120, Thailand National Biobank of Thailand, National Science and Technology Development Agency, Thailand Science Park Pathum Thani Thailand

**Keywords:** Flora of Thailand, limestone, nettle family, rhizomatous plant, Rosales, sandstone, taxonomy

## Abstract

Three new species of *Elatostema* (Urticaceae) from Thailand, *E.kaweesakii* Triyutth. & L.F.Fu, **sp. nov.**, *E.rubricaule* Triyutth. & L.F.Fu, **sp. nov.** and *E.saxatile* Triyutth. & L.F.Fu, **sp. nov.**, are newly described and illustrated. These new species can be distinguished by the presence of rhizome. *Elatostemakaweesakii* is similar to *E.atroviride*. *Elatostemakaweesakii* is a lithophyte growing in limestone crevices. It differs from *E.atroviride* by its large swollen rhizome, glabrous stem, glabrous receptacle, number of tepal in staminate flower, absence of tepal in pistillate flower, presence of staminodes in pistillate flower and smooth achene. *Elatostemarubricaule* and *E.saxatile* are found on sandstone habitats. They have distinct flattened and disk-like rhizome. *Elatostemarubricaule* is distinguished by its distinct sulcate and reddish stem with flattened and disc-like rhizome and chartaceous leaves with entire margin. *Elatostemasaxatile* resembles *E.bulbiferum* but differs by its flattened and disc-like rhizome, acute leaf apex, glabrous receptacle in pistillate inflorescences, presence of staminodes in pistillate flower, and its sandstone habitat. Descriptions, distribution, ecological and phenological data are provided.

## ﻿Introduction

*Elatostema* J.R. Forst. & G. Forst. is a large genus of Urticaceae containing several hundred species distributed throughout tropical and subtropical Asia (Christenhusz et al. 2017; Fu et al. 2021). *Elatostema* is a succulent herb normally found in evergreen forest, along stream, gorges, caves and limestone mountains (Wang 2014; Fu et al. 2017; Monro et al. 2018). Based on molecular and morphological evidences, a recent systematic study (Tseng et al. 2019) demonstrated that *Elatostema* is a monophyletic genus that includes *Pellionia* Gaudich.

The first checklist of *Elatostema* (including *Pellionia*) in Thailand documented 14 species (Yahara 1984). Since then, 20 species of *Elatostema* (including *Pellionia*) were listed by Pooma and Suddee (2014). However, a recent study involving extensive investigations has resulted in 75 species of *Elatostema* in Vietnam (Fu et al. 2019), which doubles the number of species in the previous checklist (Hiep 2005). This suggests that the species diversity documentation in Thailand was out of date due to its under-sampled status (Middleton et al. 2019).

As part of ongoing research into the flora of Thailand, the authors undertook extensive field investigations and collected three unknown specimens of *Elatostema* from the northeastern region of Thailand. After a careful comparison among specimens, it was clear that these specimens belong to the clade that includes *Pellionia* and *Elatostema* in Tseng et al. (2019), but they were distinct from other species of this clade. Therefore, we confirmed them as three new species of *Elatostema*.

## ﻿Materials and methods

Between 2016 and 2022, the authors conducted several field trips throughout Thailand. The herbarium collections from AAU, BCU, BK, BKF, C, HNU, IBK, K, L, KKU, P, PSU, QBG and SING (Thiers 2022) were examined mainly by the first author. All morphological characters were measured and compared based on dried specimens from field and herbarium collections.

## ﻿Taxonomic treatment

### ﻿1. Elatostema kaweesakii

Taxon classificationPlantaeRosalesUrticaceae

Triyutth. & L.F.Fu sp. nov.

F061AC4C-F9D0-58AF-BE41-8F3B0F2E2961

urn:lsid:ipni.org:names:77309834-1

Figs 1 , 2

#### Diagnosis.

*Elatostemakaweesakii* is similar to *E.atroviride* W.T. Wang but differed by its large swollen rhizome, staminate inflorescences with 2–7 cm long peduncle, receptacle rectangular, glabrous, staminate flower with 5 ovate tepals, pistillate inflorescences with 5–10 mm long peduncle, receptacle rectangular, glabrous, pistillate flower without tepals, staminode present and achene smooth (Table 1).

**Table 1.** Comparison of *Elatostemaatroviride*, *E.bulbiferum*, *E.kaweesakii*, *E.rubricaule* and *E.saxatile*.

Characters	*E.atroviride*	*E.kaweesakii*	*E.rubricaule*	*E.saxatile*	*E.bulbiferum*
Habitat	lithophyte in limestone soils	lithophyte in limestone crevices	lithophyte on sandstone rocks	lithophyte on sandstone rocks	lithophyte in limestone crevices
Rhizome	slender rhizome or stoloniferous	large swollen rounded rhizome	flattened, disk-like rhizome	flattened, disk-like rhizome	swollen rounded rhizome
Stem	Simple or branched, greenish, pubescent	Simple or branched, greenish, glabrous	Simple, reddish, glabrous	Simple or branched, greenish, glabrous	Simple or branched, greenish, glabrous
Nanophyll	absent	absent	absent	present	present
Leaf margin	dentate	serrate to dentate	entire	serrate	serrate
Leaf apex	acuminate	acuminate	obtuse	acute	acuminate to caudate
Staminate inflorescences: type	Capitate	Capitate	Umbellate	Umbellate	Umbellate
Staminate inflorescences: receptacle	elliptic, chartaceous, pubescent	rectangular, chartaceous, glabrous	absent	absent	absent
Staminate inflorescences: peduncle	2–5 mm long	2–7 cm long	2– 3 cm long	1–5 cm long	2–5 cm long
Staminate flower: tepal number	4	5	5	5	5
Pistillate inflorescences: type	capitate	capitate	capitate	capitate	capitate
Pistillate inflorescences: receptacle	oblong, membranous, pubescent	rectangular, chartaceous, glabrous	elliptic, membranous, glabrous	elliptic, membranous, glabrous	elliptic, membranous, pubescent
Pistillate inflorescences: peduncle	2–4 mm long, glabrous	5–10 mm long, glabrous	sessile to subsessile, glabrous	0.5–5 mm long, glabrous	0–2 mm long, glabrous
Pistillate flower: tepal number	3	absent	5	5	5
Pistillate flower: staminode	absent	3	5	5	absent
Achene	6–8 ribbed	smooth	smooth	smooth	smooth

#### Type.

Thailand. Loei: Nong Hin district, 17°7.3'N, 101°56.05'E, 360 m alt., 9 August 2022, *Triyutth. 332* (holotype KKU!; isotypes AAU!, BKF!).

#### Description.

Perennial herbs, lithophytic, monoecious, rhizomatous. ***Rhizome*** rounded, 2–20 cm in diam., brownish. ***Stems*** 15–50 cm tall, simple or branched, succulent, greenish, glabrous. ***Stipules*** 2, persistent or sometimes caducous in reproductive stage, linear or lanceolate, 4–7 × 1–3 mm, membranous, glabrous. ***Nanophylls*** absent. ***Leaves*** distichous, alternate; petiole 0.5–1 cm long, greenish, glabrous; lamina asymmetrically ovate to elliptic, 5–20 × 3–10 cm, base oblique, margin serrate to dentate, apex acuminate, chartaceous; venation pinnate, major basal lateral veins present; upper and lower surfaces greenish, glabrous, cystoliths fusiform, 0.2–0.5 mm long. ***Staminate inflorescences*** axillary, solitary, capitate, 1.5–2.0 cm in diam.; peduncle 2–7 cm long, glabrous or pubescent; receptacle rectangular, 0.6–1.0 × 0.6–1.0 cm, chartaceous, glabrous; bracts ovate, connate at base, membranous, glabrous; bracteoles lanceolate, 0.6–0.8 × 0.2–0.3 mm, membranous, pubescent. ***Staminate flowers*** 80–100 per inflorescence, sessile to subsessile, glabrous; tepals 5, ovate, 1.0–1.5 × 1.0–1.5 mm, apex acuminate, membranous, pubescent; stamens 5, filaments 1.0–1.5 mm long, anthers 0.6–0.8 mm long. ***Pistillate inflorescences*** axillary, solitary, capitate 5–10 mm in diam., peduncle 0.5–1.0 cm long, glabrous; receptacle rectangular, 6–8 × 6–8 mm, chartaceous, glabrous; bracts ovate, 0.8–1.0 × 0.5–0.6 mm, membranous, pubescent; bracteoles lanceolate, 0.4–0.6 × 0.2–0.3 mm, membranous, pubescent. ***Pistillate flowers*** 40–100 per inflorescence, sessile to subsessile, glabrous; tepals absent; staminodes 3, oblong to linear, 0.2–0.3 mm long; ovary superior, ovoid, 0.3–0.5 mm long. ***Achenes*** ellipsoid, 0.8–1.2 mm long, brownish, smooth.

#### Distribution.

Currently known only from Loei province in northeastern Thailand.

#### Ecology.

Occur in limestone crevices.

#### Phenology.

Flowering and fruiting in April–December.

#### Etymology.

This species is named in honor of Mr Kaweesak Keeratikiat, who first discovered the plants.

#### Conservation status.

This species was found in fewer than 5 locations at Nong Hin district, Loei province, Thailand. Moreover, it has a very small population and very restricted distribution, and the number of mature individuals is fewer than 250. According to IUCN (2022) this species should be assessed as Endangered (EN) under IUCN criteria B2a and D1.

#### Additional specimens examined.

Thailand. Loei: Nong Hin district, 16 December 2013, *S. Saengvirotjanapat 635* (QBG!), ibid., 20 April 2021, *Triyutth. 329* (KKU!).

#### Notes.

This species is similar to *E.atroviride* in their habitat as lithophytes growing in limestone crevices. Both species have rounded rhizome, absence of nanophyll and a similar shape of leaves. However, it differs from *E.atroviride* by several characters such as its large rounded rhizome (small size of rhizome in *E.atroviride*), staminate inflorescences with 2–7 cm long peduncle (vs 2–5 mm long), receptacle rectangular and glabrous (vs elliptic and pubescent), staminate flower with 5 tepals (vs 4 tepals), pistillate inflorescence with 5–10 mm long peduncle (vs 2–4 mm long), absence of tepals in pistillate flower (vs 3 tepals), 3 staminodes (vs staminode absent) and achene smooth (vs 6–8 ribbed).

**Figure 1.**
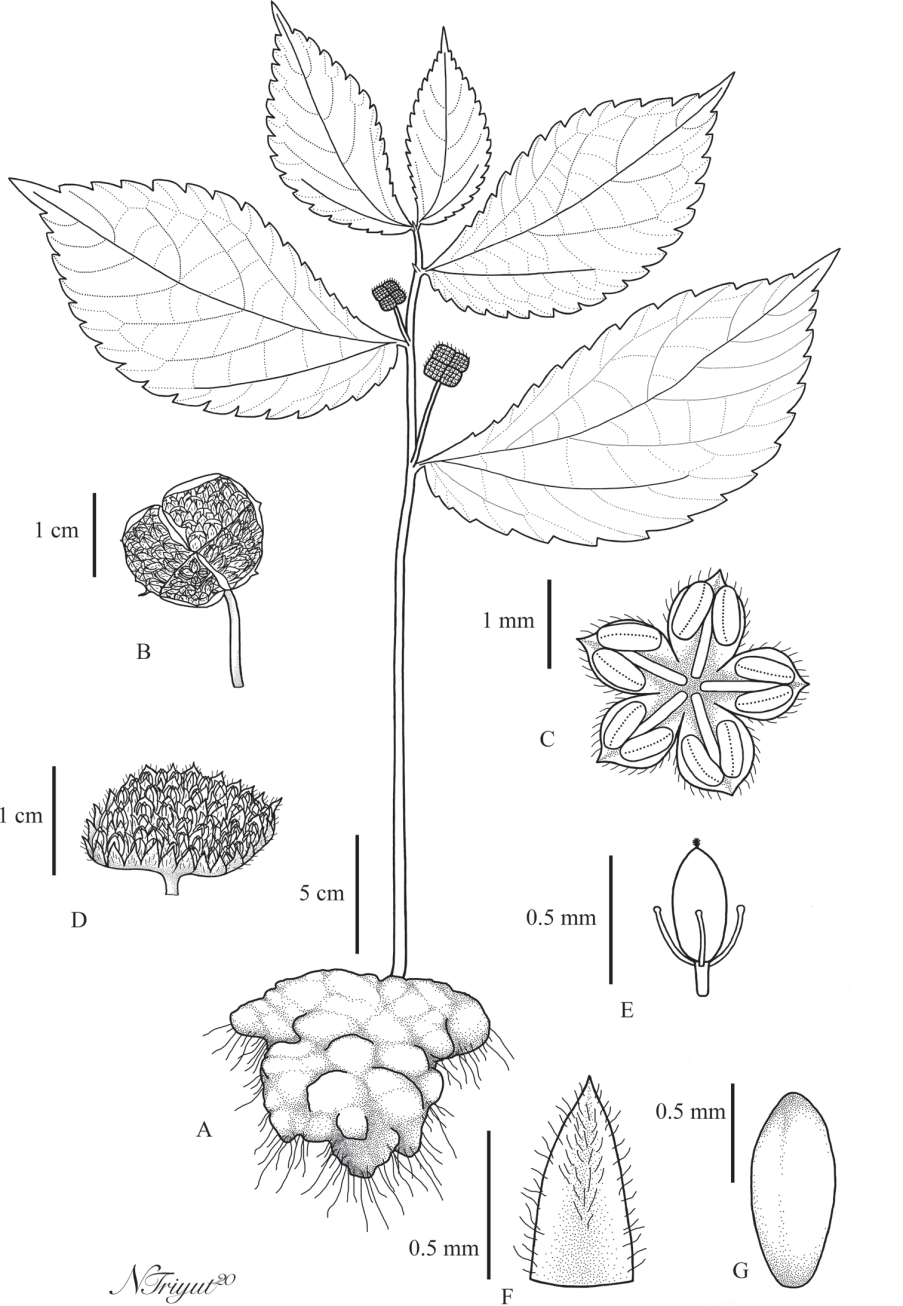
*Elatostemakaweesakii* Triyutth. & L.F.Fu, sp. nov. **A** habit **B** staminate inflorescence **C** staminate flower **D** pistillate inflorescence **E** pistillate flower **F** bracteole **G** achene (Drawn by N. Triyutthachai).

**Figure 2.**
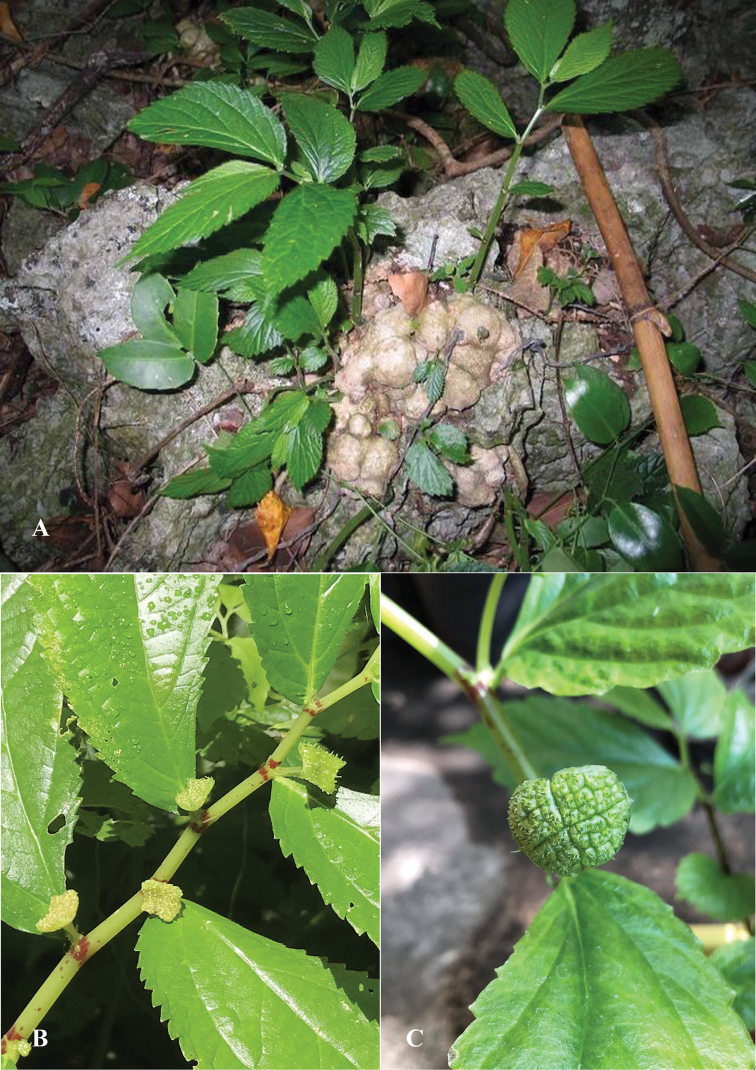
*Elatostemakaweesakii* Triyutth. & L.F.Fu, sp. nov. **A** habit and habitat **B** pistillate inflorescence **C** staminate inflorescences (Photos **A, C** by K. Keeratikiat, **B** by Triyutth).

### ﻿2. Elatostema rubricaule

Taxon classificationPlantaeRosalesUrticaceae

Triyutth. & L.F.Fu sp. nov.

2CB20138-A742-5261-85B0-8DE6D21E5BCF

urn:lsid:ipni.org:names:77309835-1

Figs 3 , 4

#### Diagnosis.

*Elatostemarubricaule* differs from other Thai *Elatostema* by its distinct sulcate and reddish stem with flattened and disk-like rhizome and chartaceous leaves with entire margin. It grows on seasonally moist sandstone rock (Table 1).

#### Type.

Thailand. Buengkan: Wat A-Hong Silawas, 18°25.47'N, 103°28.2'E, 160 m alt., 13 September 2017, *Triyutth. 201* (holotype KKU!; isotypes AAU!, BKF!, IBK!, K!).

#### Description.

Perennial herbs, lithophytic, monoecious, rhizomatous. ***Rhizome*** flattened, disk-like, 1–3 cm in diam., brownish. ***Stems*** 5–25 cm tall, simple, sulcate, succulent, reddish, glabrous. ***Stipules*** 2, persistent or sometimes caducous in reproductive stage, linear or lanceolate, 2.0–2.5 × 0.5–1.5 mm, membranous, glabrous. ***Nanophylls*** absent. ***Leaves*** distichous, alternate; petiole 0.5–2.0 mm long, reddish, glabrous; lamina asymmetrically lanceolate to elliptic, 3–5 × 0.5–1.5 cm, base asymmetrical attenuate, margin entire, apex obtuse, chartaceous; venation pinnate, major basal lateral veins absent, lateral veins 5–7 pairs; upper surface greenish, glabrous, cystolith fusiform, 0.2–0.5 mm long; lower surface cinereous, glabrous, cystoliths fusiform, 0.2–0.5 mm long. ***Staminate inflorescences*** axillary, solitary, umbellate; peduncle 2–3 cm long, glabrous; receptacle absent; bracts lanceolate, 0.8–1.0 × 0.3–0.5 mm, membranous, pubescent; bracteoles lanceolate, 0.6–0.8 × 0.2–0.3 mm, membranous, pubescent. ***Staminate flowers*** 5–10 per inflorescence; pedicel 1–3 mm long, glabrous; tepals 5, ovate to oblong, 1.0–1.5 × 1.0–1.5 mm, apex obtuse, membranous, glabrous; stamens 5, filaments 1.5–2.0 mm long, anthers 0.6–1.0 mm long. ***Pistillate inflorescences*** axillary, solitary, capitate 5–8 mm in diam., sessile to subsessile, glabrous; receptacle elliptic, 2.0–2.5 × 1.0–1.5 mm, membranous, glabrous; bracts lanceolate, 0.4–0.6 × 0.2–0.3 mm, membranous, pubescent; bracteoles lanceolate, 0.3–0.4 × 0.2–0.3 mm, membranous, pubescent. ***Pistillate flowers*** 50–80 per inflorescence; pedicel 0.5–1.0 mm long, glabrous; tepals 5, lanceolate to oblong, 0.8–1.2 × 0.2–0.4 mm, membranous, pubescent; staminodes 5, linear, 0.2–0.4 × 0.1–0.2 mm; ovary superior, ovoid, 0.3–0.5 mm long. ***Achenes*** ellipsoid, 0.5–0.8 mm long, brownish, smooth.

**Figure 3.**
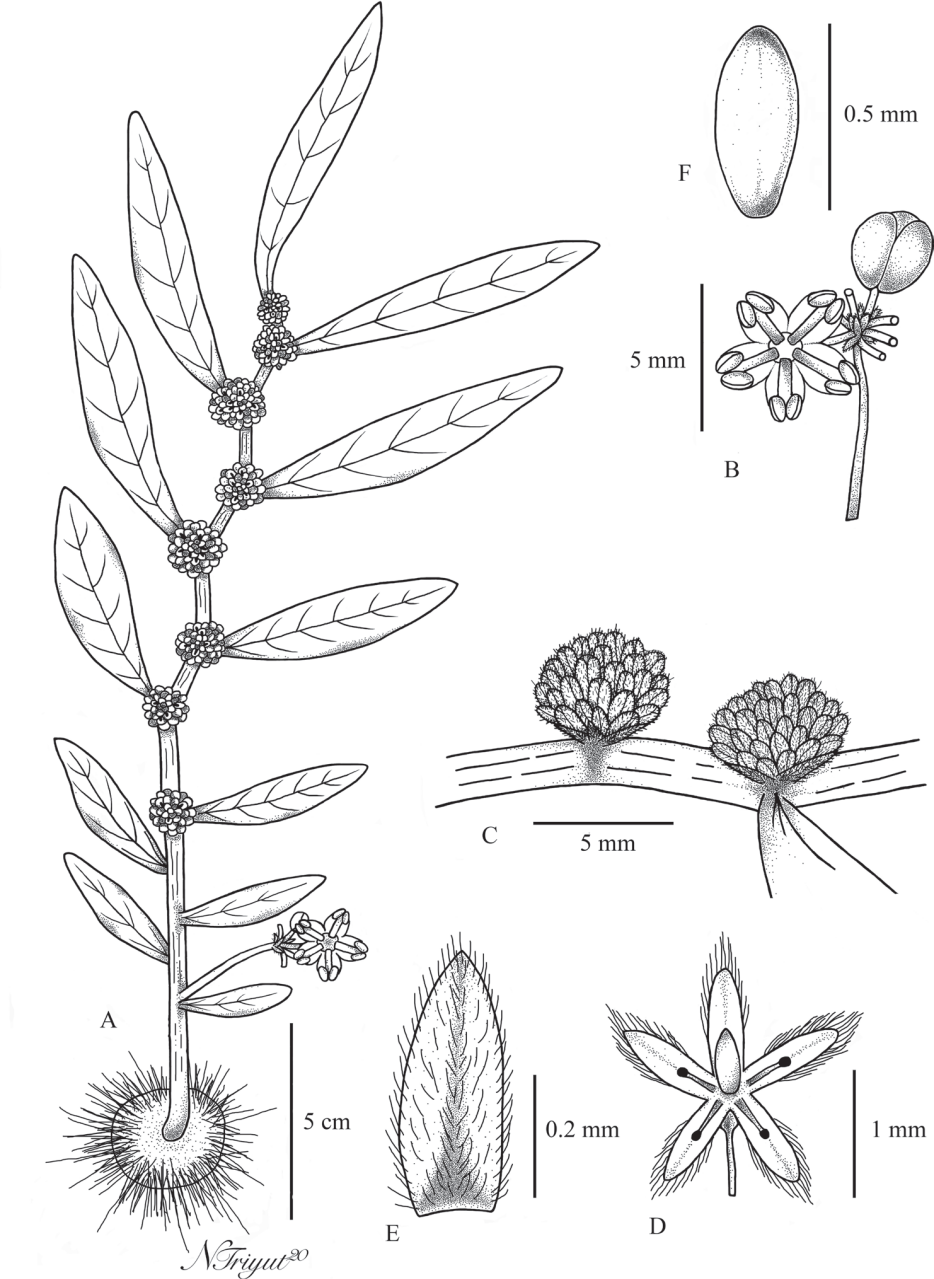
*Elatostemarubricaule* Triyutth. & L.F.Fu, sp. nov. **A** habit **B** staminate inflorescence **C** pistillate inflorescences **D** pistillate flower **E** bracteole **F** achene (Drawn by Triyutth).

**Figure 4.**
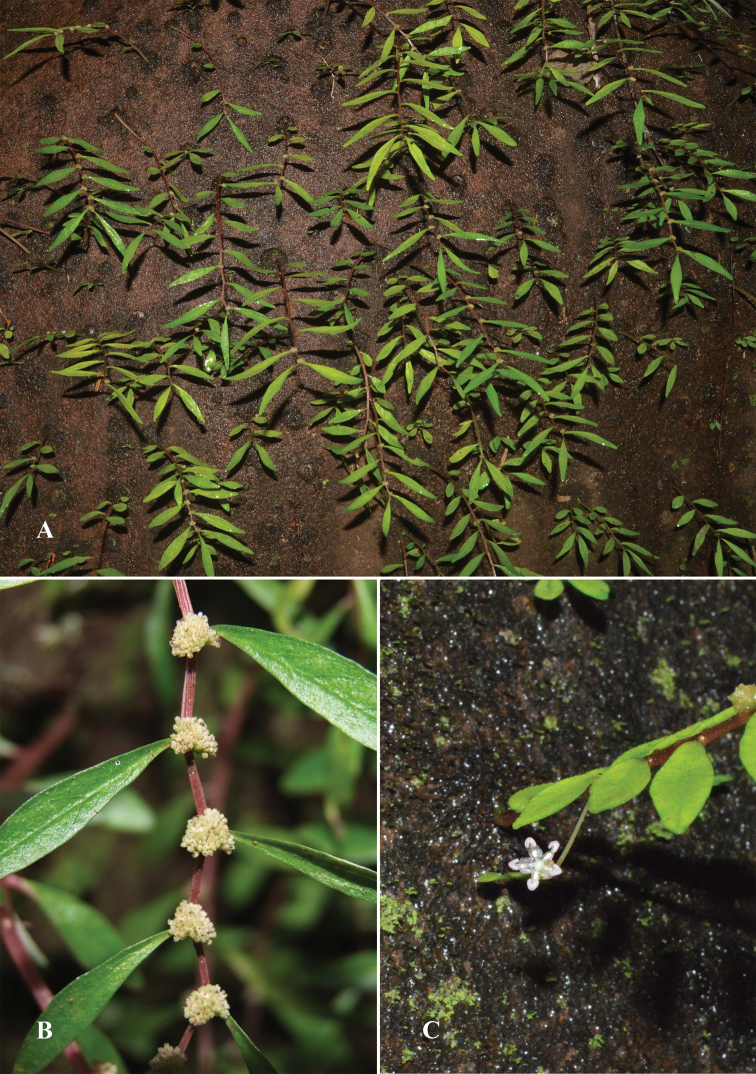
*Elatostemarubricaule* Triyutth. & L.F.Fu, sp. nov. **A** habit and habitat **B** pistillate inflorescences **C** staminate inflorescence (Photos by Triyutth).

#### Distribution.

Currently known only from the type locality in northeastern Thailand.

#### Ecology.

Occurs on seasonal moist sandstone rocks in shaded areas.

#### Phenology.

Flowering and fruiting in July–October.

#### Etymology.

Latin *ruber*, red, and *caulus*, stem, alluding to stem color of the new species.

#### Conservation status.

*Elatostemarubricaule* is currently known only from the type locality, which is not a protected area, and the number of mature individuals is fewer than 1,000. According to IUCN (2022), this species should be assessed as Critically Endangered (CR) according to criteria B1a, B2a and D1.

#### Additional specimens examined.

Thailand. Buengkan: Wat A-Hong Silawas, 18°25.47'N, 103°28.2'E, 160 m alt., 28 April 2018, *Triyutth. 269* (KKU!).

#### Notes.

This species differs from other Thai *Elatostema* by its habitat. Most species of *Elatostema* are dwelling on limestone, stream bank, gorges and caves in evergreen forest, but *E.rubricaule* was found on moist sandstone rocks near the bank of Mekong River in Buengkan Province in the northeastern part of Thailand. The most distinguished characters of this species are the flattened and disk-like rhizome, sulcate and reddish stem, chartaceous leaves with entire margin and umbellate staminate inflorescences.

### ﻿3. Elatostema saxatile

Taxon classificationPlantaeRosalesUrticaceae

Triyutth. & L.F.Fu sp. nov.

F1940737-100D-53FA-90F5-9F9830F991BD

urn:lsid:ipni.org:names:77309836-1

Figs 5 , 6

#### Diagnosis.

*Elatostemasaxatile* is similar to *E.bulbiferum* Kurz, but differs by its flattened and disk-like rhizome, receptacle of pistillate inflorescences glabrous, staminode in pistillate flower 5, acute leaf apex and its sandstone habitat (Table 1).

#### Type.

Thailand. Nakhon Phanom: Tad Kham Waterfall, 17°57.228'N, 104°9.6'E, 160 m alt., 13 September 2017, *Triyutth. 200* (holotype KKU!; isotypes AAU!, BKF!, IBK!).

#### Description.

Perennial herbs, lithophytic, monoecious, rhizomatous. ***Rhizome*** flattened, disk-like, 2–7 cm in diam., brownish. ***Stems*** 5–35 cm tall, simple or branched, succulent, greenish, glabrous. ***Stipules*** 2, persistent or sometimes caducous in reproductive stage, linear or lanceolate, membranous, glabrous. ***Nanophylls*** lanceolate to oblanceolate, 0.5–1.5 × 0.2–1.0 cm, chartaceous, glabrous, cystoliths fusiform. ***Leaves*** distichous, alternate; petiole 1–3 mm long, glabrous or puberulous; lamina asymmetrically elliptic to obovate, 1.5–7.0 × 0.5–3.0 cm, base oblique, margin serrate, apex acute, chartaceous; venation pinnate, major basal lateral veins absent, lateral veins 5–7 pairs; upper surface green, glabrous, cystolith fusiform, 0.2–0.5 mm long; lower surface greenish or cinereous, glabrous, cystolith fusiform, 0.2–0.5 mm long. ***Staminate inflorescences*** axillary, solitary, umbellate; peduncle 1–5 cm long, glabrous; receptacle absent; bracts lanceolate, 0.6–1.0 × 0.3–0.4 mm, membranous, pubescent; bracteoles lanceolate, 0.5–0.8 × 0.2–0.3 mm, membranous, pubescent. ***Staminate flowers*** 10–30 per inflorescence; pedicel 1.0–1.5 mm long, glabrous; tepals 5, ovate to oblong, 1.0–1.5 × 1.0–1.5 mm, apex obtuse, membranous, glabrous; stamens 5, filaments 1.0–1.5 mm long, anthers 0.6–1.0 mm long. ***Pistillate inflorescences*** axillary, solitary, capitate, 2–8 mm in diam., subsessile to pedunculate; peduncle 0.5–5.0 mm long, glabrous; receptacle elliptic, 2–4 mm in diam., membranous, glabrous; bracts lanceolate, 0.2–0.3 × 0.8–1.0 mm, membranous, pubescent; bracteoles lanceolate, 0.2–0.3 × 0.5–0.8 mm, membranous, pubescent. ***Pistillate flowers*** 20–50 per inflorescence; pedicel 0.5–1.5 mm long, glabrous; tepals 5, lanceolate, 0.8–1.2 × 0.2–0.4 mm, membranous, pubescent; staminodes 5, oblong, 0.1–0.2 × 0.1–0.2 mm; ovary superior, ovoid, 0.4–0.8 mm long. ***Achenes*** ellipsoid, 0.8–1.2 mm long, brownish, smooth.

**Figure 5.**
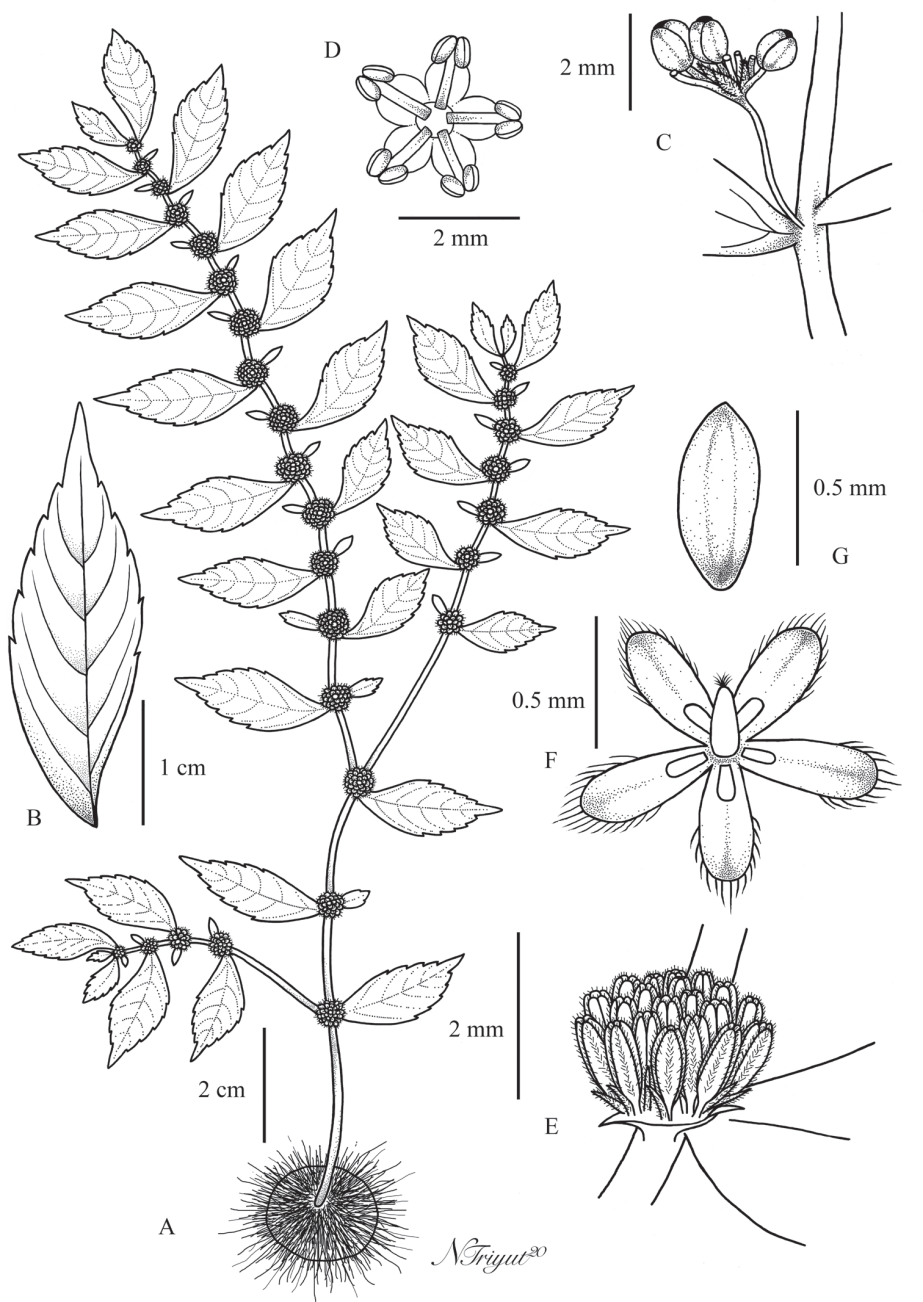
*Elatostemasaxatile* Triyutth. & L.F.Fu, sp. nov. **A** habit and habitat **B** leaf **C** staminate inflorescence **D** staminate flower **E** pistillate inflorescence **F** pistillate flower **G** achene (Drawn by N. Triyutthachai).

**Figure 6.**
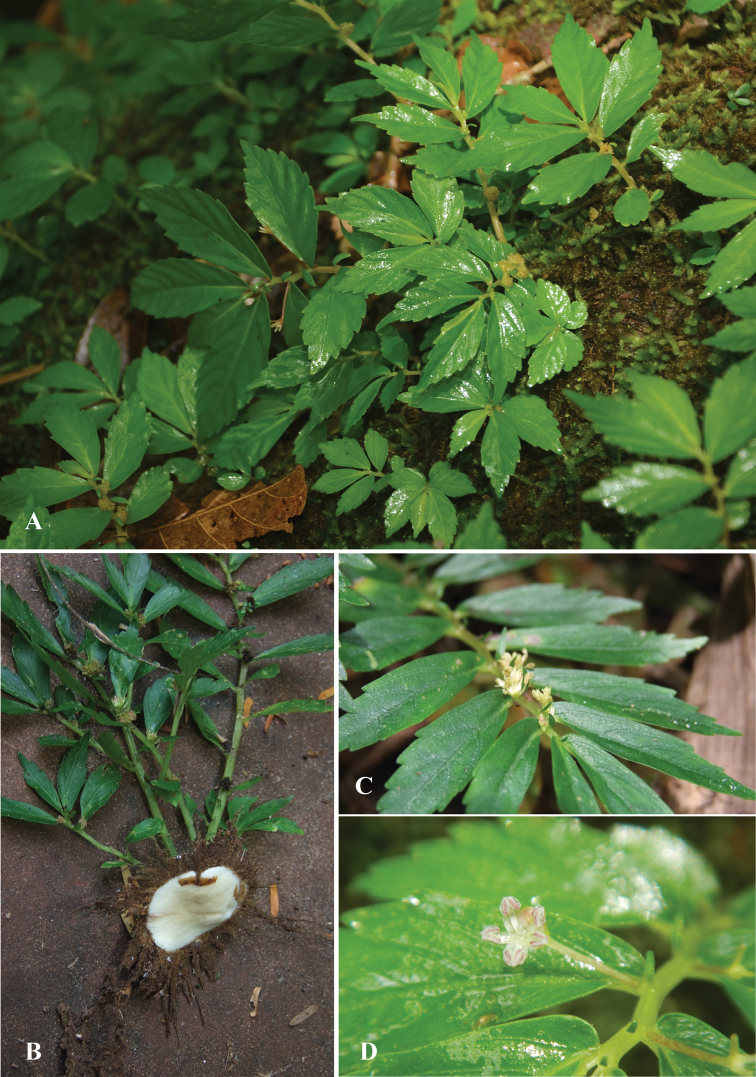
*Elatostemasaxatile* Triyutth. & L.F.Fu, sp. nov. **A** habit and habitat **B** stem and rhizome **C** pistillate inflorescences **D** staminate inflorescence (Photos by Triyutth).

#### Distribution.

Northeastern Thailand.

#### Ecology.

Occurs on seasonal moist sandstone rocks in dry evergreen forest, at 100–200 m alt.

#### Phenology.

Flowering and fruiting in May–October.

#### Etymology.

The specific epithet refers to the habitat of this plant that dwelling on the rock.

#### Conservation status.

This species was found scattered in ca. 6 locations in the Northeastern part of Thailand and the number of mature individuals is fewer than 1,000. According to IUCN (2022), *E.saxatile* should be assessed as Vulnerable (VN) according to criteria B2a and D1.

#### Additional specimens examined.

Thailand. Buengkan: Chet Si Waterfall, 14 September 2017, *Triyutth. 202* (KKU!), ibid., 27 April 2018, *Triyutth. 266* (KKU!), *267* (KKU!), Chanean Waterfall, 14 September 2017, *Triyutth. 203* (KKU!), Phu Wua, 21 May 2004, *R. Pooma et al. 4201* (BKF!), *4202* (BKF!), *4191* (BKF!), ibid., 28 December 2011, *M. Norsaengsri* & *N. Tathana 8707* (QBG!), ibid., 13 July 2016, *Triyutth. 97* (KKU!), *98* (KKU!), Phu Sing, 26 August 2001, *R. Pooma et al. 2694* (BKF!), Phu Tok, 27 April 2018, *Triyutth. 268* (KKU!), Phu Tok Noi, 22 June 1995, *C. Niyomdham 4448* (BKF!), ibid., 21 June 1997, *C. Niyomdham 5052* (BKF!), Wat Tham Phra, Phu Wua, 20 May 2014, *S. Sirimongkol et al*. *593* (BKF!); Nakhon Phanom: Tad Kham Waterfall, 25 August 2001, *R. Pooma et al. 2652* (BKF!), ibid., 13 September 2017, *Triyutth. 199* (KKU!).

#### Notes.

*Elatostemasaxatile* was found growing on the sandstone rocks in Buengkan and Nakhon Phanom Provinces, in the Northeastern part of Thailand. This species is similar to *E.bulbiferum* in the presence of rhizome, presence of nanophyll and umbellate staminate inflorescences, but differed by its flattened and disc-like rhizome (vs rounded rhizome in *E.bulbiferum*), receptacle of pistillate inflorescences glabrous (vs pubescent), staminode in pistillate flower 5 (vs absent), acute leaf apex (vs acuminate to caudate apex). Moreover, *E.saxatile* was found on sandstone while *E.bulbiferum* was found on limestone substrates.

## Supplementary Material

XML Treatment for Elatostema kaweesakii

XML Treatment for Elatostema rubricaule

XML Treatment for Elatostema saxatile
